# Foreign National Patients in German Prison Psychiatry

**DOI:** 10.3389/fpsyt.2019.00988

**Published:** 2020-02-14

**Authors:** Britta Neumann, Thomas Ross, Annette Opitz-Welke

**Affiliations:** ^1^ Institute of Forensic Psychiatry, Charité University of Medicine Berlin, Berlin, Germany; ^2^ Forensic Psychiatry and Psychotherapy, Reichenau Psychiatric Center and University of Ulm, Ulm, Germany

**Keywords:** prison psychiatry, high security hospital, diminished culpability, compulsory treatment, foreign national prisoners, citizenship

## Abstract

**Introduction:**

Over the past few years, the share of foreign national prisoners in the European and American justice systems has increased at a disproportionately high rate, yet studies on mental health issues among this diverse group are rare. Recent research suggests a range of factors leading to mental health vulnerability in foreign national prisoners, including language barriers, isolation, cultural misunderstanding, and legal standing. Relevant findings of topic-related studies indicate that under-referral to mental health services due to missed or misinterpreted symptoms is a major risk for foreign national prisoners.

**Aims:**

We aimed to investigate the disparities regarding the percentage of foreign national patients who were treated in high-security hospitals compared to the psychiatric ward of prison hospitals—after adjusting for diagnosis, age, marital status, and substance abuse. We hypothesized that foreign national patients were underrepresented in compulsory, high-security mental health care. We also aimed to explore citizenship-related institutional disparities concerning diagnoses and self-harmful behavior.

**Method:**

From 2010 to 2015, data collected from high-security hospitals in the federal state of Baden-Wurttemberg and the psychiatric ward of a Berlin prison hospital was evaluated by comparing nationality, diagnosis, and self-harm using Fisher’s exact test and χ²-test. The odds ratios for citizenship-related differences in diagnosis and institution of treatment were evaluated by using logistic regression.

**Results:**

Mentally ill foreign national patients were significantly less likely to be treated in high-security hospitals rather than prison hospital psychiatry (adjusted for diagnosis, age at admission, marital status, and substance abuse; adjusted OR = 0.5). Foreign nationals and Germans in prison hospital psychiatry showed no significant disparities in diagnosis; however, in high-security hospitals, foreign nationals were more likely to have been diagnosed with schizophrenia/psychotic or neurotic/stress-related disorders and were less likely to have been diagnosed with personality disorders than German patients. Additionally, foreign nationals were more likely to commit self-harm than Germans in prison hospital psychiatry, but significant citizenship-related differences could not be verified in high-security hospital patients.

**Conclusion:**

Treatment conditions of foreign national patients in prison psychiatry must be improved. To achieve this, the psychiatric assessment and (mental) health-related aspects of these patients should be further investigated.

## Introduction

In increasingly multicultural societies, the share of foreign national prisoners has grown at a disproportionately high rate over the past few years. In England and Wales, for example, foreign nationals accounted for approximately 9.4% (1, 2) of the general population and 12% of the overall prison population in 2017 (3). As further illustrated by a UK Prison Service Journal published in 2013, the number of foreign national prisoners increased by 93% between 2000 and 2012, compared to a 24% increase of British nationals (4). In the United States, non-US citizens comprised 7.2% of the general population (5) and over 21% of all federal prisoners in 2016 (excluding persons detained by the US Department of Homeland Security) (6). In Germany, 11.6% of the general population (7) and 30.1% of prisoners were foreign nationals in 2017 (8). Van Kalmthout et al. (9) stated that foreign nationals accounted for more than 20% of all European prisoners and according to the UNODC Handbook of Prisoners with Special Needs (10) and the World Prison Brief (11) foreign prisoners are significantly overrepresented in some non-Western countries as well.

Offenses related to immigration policy seem to partially explain this inequality in the justice system (12, 13), yet disparities in court sentencings for foreign nationals might also be at play (4, 10). Light et al. (14) recently revealed higher incarceration rates and longer sentencing periods for non-US citizens, even after adjusting for race and ethnicity as potential confounding factors.

The rising percentages of foreign nationals in the penal system has raised questions about their health conditions. Multiple sources indicate serious mental health issues among ethnic-minority and immigrant prisoners (15–23). Recent research suggests that the majority of factors leading to mental health vulnerability among prisoners, such as language barriers, isolation, cultural misunderstanding, and legal standing, are even more salient for foreign national prisoners (4, 12, 13, 24–26).

The principles of treatment for mentally ill offenders have been established in the legal systems of many Western countries. Offenders with a certain level of diminished responsibility may be compulsorily admitted to psychiatric care instead of an ordinary sentence, whereas criminally responsible offenders often receive the requisite psychiatric treatment during or prior to serving a prison sentence (27, 28). The German legal system involves a similar means of treating mentally ill offenders (29, 30). An offender with a certain level of diminished responsibility may be sentenced to high-security hospitals by law (§ 63 StGB, § 64 StGB) whereas inpatient mental health care for regular prison inmates is provided by physicians in prison hospitals located on prison premises if accessible (29, 31–33). Regardless, it is well understood that care in prison psychiatric wards is far less intense than that of high-security hospitals (34, 35).

Research in this field suggests that the conditions under which prison psychiatric health care is offered or compulsory treatment is imposed may place certain minority groups at a disadvantage. In their systematic review of 26 studies, Spinney et al. (36) revealed racial disparities in the US justice system, with Black and Hispanic juveniles referred to mental health and substance abuse programs less often than their White counterparts. Steadman et al. (37) found ethnic disparities among referrals to US mental health courts—courts designed “for persons with mental illness that were in part created to divert this population from jail/prison into community treatment” (38)—with non-Whites referred at a lower rate. Forrester et al. (39) stated that foreign nationals in a London prison were under-referred to mental health in-reach teams—originally developed to provide community-equivalent mental health services for prisoners (40, 41)—which raised “questions about the culturally appropriate ways in which they are advertised and delivered”.

Recent studies implicate that citizenship-related barriers might be held responsible for treatment disparity: Sen et al. (13) suggested that foreign national prisoners in England and Wales under-accessed mental health care due to factors related to applying for these services, such as a specific residency and prior registration with a general practitioner (26, 42). In the Netherlands, Vinkers et al. (43) pointed out that while compulsory admission to psychiatric hospitals was higher among non-nationals, conditional admission to penitentiaries—which is only offered to patients who are considered compliant—was lower. In Germany, Hoffmann (18) discovered that drug-abusing immigrants were rarely admitted to detoxification therapy in high-security hospitals, presumably due to language barriers. The author partially attributed his findings to the high deportation rate of immigrant offenders charged with violating the legislation on narcotics.

In addition, other studies indicate that missed or misinterpreted symptoms of mental disorders among ethnic-minority, immigrant, and foreign national prisoners might aggravate symptoms and impact self-harmful behavior. By evaluating data from a New York City jail, Kaba et al. (19) showed that Hispanic and Black prisoners with mental disorders remained undiagnosed significantly longer than White prisoners. Furthermore, non-White prisoners who began receiving mental health services at a later stage were more likely to be compulsorily admitted to solitary confinement which is considered to be associated with committing self-harm (44). In England and Wales, Borrill and Taylor (45) outlined that in 2007, foreign national prisoners accounted for 28% of all self-inflicted deaths, although this population only accounted for 16% of the prison inmates investigated. The authors stated that trauma symptoms had increased the vulnerability to suicide and that these patients had mainly received antipsychotic treatment instead of guideline-based therapy.

## Aims and Hypotheses

In this study, we aimed to identify the institutional disparities regarding the distribution of national and foreign national patients. We hypothesized that foreign national patients were more likely to enter prison hospital psychiatry than high-security hospitals, after adjusting for diagnosis, substance abuse, marital status, and age at admission. Additionally, we suspected that there were citizenship-related disparities concerning diagnosis in both institutions, after adjusting for the same variables. We further hypothesized that significantly more foreign national patients had exhibited self-harmful behavior compared to German patients in prison hospital psychiatry but not in high-security hospitals.

## Materials and Methods

### Data

The dataset for the prison hospital population was sourced from the psychiatric ward of the Berlin prison hospital (JVKB), which is located on prison premises, covering a total of 572 incarcerated males requiring inpatient mental health care between 2010 and 2015. Within the framework of administration, routine data concerning the penal, sociodemographic, and clinical aspects of the prison population were recorded and entered into the hospital database.

Every patient was assigned a unique identification number, which was derived from the prisoner’s name and date of birth and subsequently encrypted. Since hospital visitations disrupt regular incarceration, the monitoring of symptoms was inconsistent. Each admission to the psychiatric ward was recorded using a new entry in the database system, potentially including a new diagnosis. We registered multiple admissions in 91 patients, 63 of these patients were admitted twice and 28 of these patients multiple times (the rates of admissions ranging from three to seven times). To prevent overweighting of those who were repeatedly admitted, we cumulated the data. In the 19 cases where the main diagnosis had changed, we considered the last-assigned main diagnosis in our calculations.

Marital status and age always related to the patient’s status at initial admission. To estimate the percentage of patients with substance abuse problems, all diagnoses, including secondary diagnoses, given during all admissions of each patient were evaluated. Incidences of self-harm were recorded as a dichotomous variable (self-harm/no self-harm) in each admission. When cumulating data of patients with multiple admissions, we categorized self-harm as positive as soon as it was recorded at least once.

To ensure the validity and reliability of the clinical data assessment tool, all entries (categories, sub-categories, single variables) are explained to all staff members working in the Berlin prison hospital including detailed instructions on the meaning and content of the items. The majority of the collected data is derived from routine data which is also recorded by statutory health insurances.

The dataset for the high-security hospital population was sourced from eight high-security hospitals in the federal state of Baden-Wurttemberg. Routine data concerning the penal, sociodemographic, and clinical characteristics of 1,883 male patients—admitted to high-security hospitals from 2010 to 2015—was entered into a cross-hospital database and subsequently evaluated. Patients who had been transferred from external high-security hospitals or who were on revocation were not taken into consideration, as the actual date of admission was inaccessible. Every patient was assigned a unique identification number, which was derived from the identification numbers of hospital interns. To ensure the merging of data for patients who had changed facilities during treatment and consequently received a new number, we also gathered a combination of birthplace, birthdate, and date of judgment for each patient. After initial admission, data on each patient was consistently maintained and incidences were annually documented. The diagnosis considered in our calculations refers to the last recorded main diagnosis (diagnoses in high-security hospitals are rarely altered). Marital status and age always related to the patient’s status at admission. Substance abuse and self-harm were recorded as dichotomous variables (yes/no) and categorized as positive as soon as they were recorded in at least one of the annual entries of the respective patient.

To ensure the validity and reliability of the clinical data assessment tool, all entries (categories, sub-categories, single variables) are explained in a glossary accessible to all forensic therapists working in forensic psychiatric units across the State of Baden-Wurttemberg. The glossary has detailed instructions on the meaning and content of the items, guiding data-managers through otherwise difficult to rate items. This is to make sure that therapists understand the same thing by each variable. The data were entered by the patient’s principal therapist, reviewed by the chief medical officers, and anonymized. Thus, no researcher was or has been able to identify individual patients using the dataset. The data was collected and computed in accordance with the WMA declaration of Helsinki.

### Methods

For our study, we aimed to compare the most common means of treating mentally ill offenders in Germany. The so-called *Maßregelvollzug* is comparable to high-security hospitals in other western countries and therefore referred to as such. A further classification into low-, medium- or high-security hospitals is hardly relevant in the German legal system.

To allow for standardized classification, citizenship was used as a distinguishing characteristic. Migration background was not considered, since that information was not accessible for all patients. Patients with current German citizenship were considered nationals, including patients with dual nationality. It should be noted that this approach led to very heterogeneous groups as the patients’ former citizenships or the countries of origin were not taken into consideration. Patients with unclear citizenship status (i.e. five in Berlin; 0.9%) were excluded from our calculations, leaving a total number of 567 patients in prison hospital psychiatry and 1,883 patients in high-security hospitals.

Due to individual institution-related regulations, the structure of the data-bases differed considerably. In order to achieve comparability of the investigated variables, the required content was firstly extracted and subsequently inserted in respective overview tables.

The data was then analyzed *via* logistic regression, using the Wald test and the likelihood ratio to determine significance. The crude and adjusted odds ratios were evaluated using a 95% confidence interval. First, we performed logistic regression to identify the odds ratio of mentally ill foreign national patients who would be treated in prison hospital psychiatry rather than in high-security hospitals, after adjusting for diagnosis, age, and marital status at admissions and substance abuse. Additionally, we performed the same analyses on patients with schizophrenia and psychotic disorders, affective mood disorders, and personality disorders.

We then applied similar procedures to investigate significant citizenship-related disparities in diagnosis across both institutions, adjusting for age and marital status at admission, as well as substance abuse. Further, we used χ²-test to evaluate significant citizenship-related disparities in diagnoses and Fisher’s exact test to evaluate significant institutional disparities in percentages of foreign national patients and significant citizenship-related disparities in self-harmful behavior.

Statistical analyses was performed by using R version 3.5.1.

All data was obtained during routine administration and sufficiently anonymized. Approval for the research has been obtained from the local ethics committee at Charité, Berlin University of Medicine.

## Results

**Table 1** shows the absolute numbers and percentages of German and foreign national patients treated in prison hospital psychiatry and high security hospitals.

**Table 1:** Citizenship of patients in high security hospitals and prison hospital psychiatry.

	Prison hospital psychiatry		High security hospitals		*P* (Fisher's exact test)
	n = 567		n = 1,883		<0.001
German	329	(58.0%)	1,449	(77.0%)	
Non-German	238	(42.0%)	434	(23.0%)	

**Table 2A** displays disparities in diagnosis related to the nationalities of patients treated in prison hospital psychiatry. **Table 2B** exhibits the adjusted odds ratios which predict the probability of receiving the respective diagnosis. The type of disorder did not differ significantly between foreign national and German patients in prison hospital psychiatry after adjusting for age at admission, marital status, and substance abuse.

**Table 2A:** Main Diagnoses in prison hospital psychiatry patients.

	German		Non-German		*P* (χ²-test)
	n = 323^1^		n = 233^1^		0.094
Substance abuse disorders	20	(6.19%)	11	(4.72%)	
Schizophrenia and psychotic disorders	150	(46.4%)	127	(54.5%)	
Affective mood disorders	27	(8.36%)	20	(8.58%)	
Neurotic, stress-related disorders	87	(26.9%)	63	(27.0%)	
Personality disorders	24	(7.43%)	7	(3.00%)	
Other	15	(4.64%)	5	(2.15%)	

^1^Eleven patients with missing diagnoses were excluded from analyses.

**Table 2B:** Odds ratios (95% CI) for diagnoses in foreign national patients compared with German patients in prison hospital psychiatry.

Variable	Unadjusted	Adjusted^1^	*P* (Wald test)
Diagnosis, ref. = F1			
Psychotic disorders	1.46 (0.67, 3.18)	1.51 (0.67, 3.38)	0.316
Mood affective disorders	1.28 (0.5, 3.28)	1.22 (0.45, 3.26)	0.699
Neurotic/stress-related disorders	1.25 (0.56, 2.81)	1.01 (0.43, 2.36)	0.981
Personality disorders	0.5 (0.16, 1.55)	0.46 (0.15, 1.49)	0.197
Other	0.58 (0.16, 2.02)	0.57 (0.16, 2.11)	0.404

^1^Adjusted for age, marital status and substance abuse.

**Table 3A** shows the last assigned main diagnosis in foreign national patients compared with German patients treated in high-security hospitals. **Table 3B** illustrates the odds ratios which predict the probability of receiving the respective diagnosis. Foreign national patients were more likely to have been diagnosed with schizophrenia and psychotic disorders (adjusted OR = 2.06), neurotic and stress-related disorders (adjusted OR = 6.06), and less likely with personality disorders (adjusted OR = 0.33) compared to the reference value (substance abuse disorders) than German patients after adjusting for age at admission, marital status, and substance abuse.

**Table 3A:** Main Diagnoses in high security hospital patients.

	German		Non-German		*P* (χ²-test)
	n = 1,445^1^		n = 431^1^		< 0.001
Substance abuse disorders	872	(60.3%)	230	(53.4%)	
Schizophrenia and psychotic disorders	370	(25.6%)	168	(39.0%)	
Affective mood disorders	29	(2.01%)	8	(1.86%)	
Neurotic, stress-related disorders	2	(0.14%)	5	(1.16%)	
Personality disorders	85	(5.88%)	7	(1.62%)	
Other	87	(6.02%)	13	(3.02%)	

^1^Seven patients with missing diagnoses were excluded from analyses.

**Table 3B:** Odds ratios (95% CI) for diagnoses in foreign national patients compared with German patients in high security hospitals.

Variable	Unadjusted	Adjusted^1^	*P* (Wald's test)	*P* (LR-test)
Diagnosis, ref. = F1				< 0.001
Psychotic disorders	1.74 (1.37, 2.19)	2.06 (1.58, 2.7)	< 0.001	
Mood affective disorders	1.06 (0.48, 2.34)	1.02 (0.45, 2.34)	0.956	
Neurotic/stress-related disorders	9.56 (1.84, 49.6)	6.06 (1.11, 33.07)	0.038	
Personality disorders	0.31 (0.14, 0.69)	0.33 (0.15, 0.73)	0.006	
Other	0.58 (0.32, 1.05)	0.66 (0.35, 1.27)	0.216	

^1^Adjusted for age, marital status, and substance abuse.

**Table 4** presents the adjusted odds ratios which predict the probability of receiving mental health care in high-security hospitals rather than prison hospital psychiatry for foreign nationals. After adjusting for diagnosis, age and marital status at admission, and substance abuse, we found that foreign national patients were half as likely (adjusted OR = 0.5, *P* < 0.001) to be treated in high-security hospitals than in prison hospital psychiatry. Similar results were found for foreign national patients with schizophrenia and psychotic (adjusted OR = 0.57), affective (adjusted OR = 0.18), and personality disorders (adjusted OR = 0.29).

**Table 4:** Odds ratios (95% CI) for treatment in high security hospitals in foreign national patients compared with German patients.

Variable	Unadjusted	Adjusted^1^	*P* (Wald's test)	*P* (LR-test)
All diagnoses	0.41 (0.34, 0.5)	0.5 (0.39, 0.65)	< 0.001	< 0.001
Psychotic disorders	0.54 (0.4, 0.72)	0.57 (0.41, 0.77)	< 0.001	< 0.001
Mood affective disorders	0.37 (0.14, 0.99)	0.18 (0.05, 0.63)	0.008	0.003
Personality disorders	0.28 (0.09, 0.88)	0.29 (0.09, 0.94)	0.038	0.041

^1^Adjusted for age, marital status, and substance abuse.

We further discovered that, compared to German patients, a significantly greater number of foreign nationals who were treated in prison hospital psychiatry had committed self-harm (see **Table 5A**, *P* < 0.005); however, no significant disparities related to citizenship were found in high-security hospitals (*p* = 0.177) (see **Table 5B**).

**Table 5A:** Self-harm in foreign national and German patients in prison hospital psychiatry.

	German patients	Foreign national patients	*P* (Fisher's exact test)
No self-harm	318 (96.7%)	216 (90.8%)	0.005
Self-harm	11 (3.34%)	22 (9.24%)	

**Table 5B:** Self-harm in foreign national and German patients in high security hospitals.

	German patients	Foreign national patients	*P* (Fisher's exact test)
No self-harm	1,398 (96.5%)	425 (97.9%)	0.177
Self-harm	51 (3.52%)	9 (2.07%)	

## Discussion

Compared to their share among the general population, foreign nationals are clearly overrepresented in both institutions. In prison hospital psychiatry, foreign nationals accounted for 42% of all patients, which is significantly higher than the average of 33.2% foreign nationals in the Berlin penal system (not including remand prisoners) and 13.6% in the general population, as reported in the reference period (7, 46). In high-security hospitals, however, foreign nationals accounted for 23% of all patients, indicating an underrepresentation compared to the percentage of foreign nationals in the Baden-Wurttemberg penal system (average of 35%) (47), yet an overrepresentation compared to the general population (average of 12.1%), as reported in the reference period (7).

In discussions of the high rates of ethnic minorities, immigrants, or foreign nationals in prison psychiatry compared to community-based mental health care, the factors of deinstitutionalization, culturally influenced behavior patterns, and the drawbacks of migration and deprivation are often referenced (15, 18, 27, 48). Among these groups, access to voluntary psychiatric treatment services is scarce, especially non-acute outpatient services, supposedly owing to culturally influenced perceptions and assessments of psychiatric symptoms, the patient’s lack of confidence in the foreign country, insufficient experience in medicating these patients among public healthcare professionals, and the social marginalization experienced by patients (20, 22, 49–52). When “forensification” is present, referring to the failure to adequately treat severely mentally ill patients in general psychiatry, thus resulting in their incarceration and subsequent treatment in forensic psychiatric institutions (53), Leese et al. (21) stated that the consequential “revolving-door” practice might be even more accurate when describing the mental health care received by ethnic-minority patients.

In our study, the clearest disparity in the treatment of mentally ill foreign national patients is related to the institution providing the mental health services. Compared to German patients, we found that foreign nationals were half as likely to be treated in high-security hospitals rather than in prison hospital psychiatry, after adjusting for diagnosis, age at admission, marital status, and substance abuse (adjusted odds ratio = 0.5). The odds ratios were even lower for foreign nationals with affective mood and personality disorders.

In Germany, referrals to high-security hospitals are based on a psychiatric assessment conducted during the prosecution of a serious crime (28, 31). The treatment setting of patients requiring intensive treatment is therefore primarily bound to the outcome of the court procedure (32, 34, 35). This could imply that foreign nationals commit less serious crimes (e.g. immigration-related offenses), as we did not adjust for this variable due to the limited amount of data available.

Our findings revealed that foreign nationals in prison hospital psychiatry were at a significantly higher risk of committing self-harm than German patients, whereas we observed no significant differences concerning citizenship in high-security hospitals. This gives rise to the assumption that the symptoms of mental disorders displayed by foreign national patients, either before or during trial and also in custody, remain undetected or are susceptible to misinterpretation. Symptoms that are overlooked or misinterpreted could prevent the appropriate referral to mental health care. In a study conducted by Borrill and Taylor (45), the authors evaluated the self-inflicted death of 20 foreign national patients in England and Wales in 2007. Two of the outlined risk factors were difficulties expressing health symptoms due to language barriers and insufficient treatment of trauma patients.

Priebe et al. (22) conducted several surveys evaluating the opinions of healthcare professionals on the current state of health care for migrants across 16 European countries. Eight problem areas were identified, of which five may be easily transferred to the general psychiatric assessment of foreign nationals:

1) Language and
2) Cultural barriers were commonly reported and considered relevant in the misdiagnosing of ethnic-minority patients.
3) Different understandings of illness (and treatment) are considered fundamental to health care. While professionals usually apply a scientific approach, this may diverge greatly from culture-specific approaches to etiopathology.
4) A further issue is the impact of socioeconomic factors including deprivation and traumatic experiences. These factors might greatly influence the formation of (psychiatric) illnesses and, if not recorded in the patient’s anamnesis, distort the assessment of symptoms.
5) Lack of trust in staff members was also commonly reported, which impeded patient assessment.

Additionally, mental disorders in patients without previous community-based treatment might remain undetected, as this data is often collected during the first health assessment of prisoners (54, 55).

However, it should also be considered that foreign nationals might be more susceptible to prison circumstances (e.g. elevated risk of isolation, deportation issues), resulting in higher admittance to prison hospital psychiatry and incidences of self-harm (45, 56).

Although there were no significant differences in prison psychiatric diagnosis related to citizenship, after adjusting for marital status, age at admission, and substance abuse, foreign nationals treated in high-security hospitals were more likely to have been diagnosed with neurotic/stress-related disorders (though numbers were very low in general) and schizophrenia/psychotic disorders, yet were less likely to have been diagnosed with personality disorders. Considering the pre-trial assessment, this could imply that ethnic-minority patients might initially be diagnosed with neurotic/stress-related disorders—disorders that are usually not suitable for alternative treatment in high-security hospitals (29)—and schizophrenia/psychotic disorders. Al-Rousan et al. (54) recently pointed out that the mean time intervals between the start of incarceration and the first diagnosis for inmates in Iowa varied broadly depending on the disorder diagnosed. While the mean interval to first diagnosis of psychotic disorders was approximately 14 months, the first diagnoses of depression, PTSD, and personality disorders tended to occur at 26, 21, and 29 months, respectively. It appears that the symptoms of psychotic disorders are more evident and thus they could be less affected by the citizenship-related barriers to assessment.

Some studies have indicated higher levels of psychotic disorders in ethnic, immigrant, or foreign national than in national offenders and associated these disorders with a higher rate of compulsory psychiatry treatment. According to the authors, these discoveries might partly be due to incomplete explorations and understandings of language barriers and cultural knowledge (18, 27, 43, 48, 57–59). In the United States, for example, Perry et al. (27) revealed that African Americans were far more likely to receive psychotic diagnosis and as a consequence, were found not criminally responsible by court. The authors declared that this might have positive effects on the patients’ mental health, yet also stated that treatment due to misdiagnosis could be ineffective and stigmatizing.

In Canada, Kirmayer et al. (60) revealed serious deficits in the diagnostics and treatment of ethnic-minority patients, including immigrants and refugees, by applying an expanded version of the DSM-IV Cultural Formulation—a model “assessing cultural identity, cultural explanations of the illness, cultural factors related to the psychosocial environment and levels of functioning, cultural elements of the clinician–patient relationship, and the overall impact of culture on diagnosis and care” [(61), p. 271]. Adeponle et al. (57) demonstrated that, after applying the DSM-IV Cultural Formulation, a substantial percentage of patients initially diagnosed with a psychotic disorder were re-diagnosed with a non-psychotic disorder. As Gara et al. (58) pointed out, misdiagnosis in these patients might worsen treatment response and lower treatment expectations.

The type and severity of disorder diagnosed by a psychiatric expert witness usually plays an essential role when considering high-security hospital treatment for mentally ill offenders (27–29, 31, 43, 48). Research suggests that ethnic-minority patients are susceptible to stereotyping by physicians, psychiatrists, and judges, which implies that the complexity of their psychiatric assessment might be reduced to prejudiced assumptions about patient adherence to treatment recommendations and to associating signs of mental illness with personality traits rather than actual health disorders (19, 27, 43, 62–65). In a UK study, Mikton and Grounds (63) searched for disparities in the diagnosing of personality disorders by forensic psychiatrists working with different ethnic groups in England and Wales. Their results indicated that antisocial personality disorder was less often diagnosed in African-Caribbean patients compared to White patients. The authors speculated that this was attributed to cross-cultural clinical judgment bias or ethnically insensitive diagnostic testing. Similar measures might apply to pre-trial assessments, as personality disorders were significantly underrepresented in foreign national patients receiving treatment at high-security hospitals.

### Limitations

In our study, we divided patients into groups of nationals and non-nationals according to their current citizenship. Former citizenships or countries of birth were not taken into consideration, as these were not accessible for all patients, thus limiting the results of our study to a certain extent.

It should be noted that no female patients were treated in the Berlin prison hospital, hence female patients treated in high-security hospitals in Baden-Wurttemberg were excluded from our study. Therefore, the outcome of our study may only be considered valid for male patients. Further studies should be conducted to determine the treatment conditions of female patients.

As indicated above, high-security hospitals and prison hospital psychiatry differ substantially depending on admission, period of treatment, and patient records. While patients in high-security hospitals are consistently monitored for years, prison psychiatric patients are only assessed during their irregular and temporary visits to the hospital ward, which does not provide a clear picture of incidents occurring or symptoms displayed during regular incarceration.

This raises the question as to whether multiple admissions to prison hospital psychiatry should be individually compared, thus overweighting patients who are admitted more frequently, or whether the data of each patient should be merged. For reasons of comparability, we decided to follow the latter option, which led to a conflict regarding diagnosis. Since each admission created a new record of administrative data, the patient’s diagnosis was potentially altered each time (this was the case in 19 patients). To prevent overvaluation of preliminary diagnoses we decided to focus on each patient’s last-assigned main diagnosis. In order to allow better comparison between both institutions, age, and marital status were recorded on initial admission, therefore leading to a discrepancy in the date of the recording of the different variables.

These limitations have influenced our direct comparison between both systems to a certain extent. Furthermore, each institution uses their own database, which are subject to variation due to differing in-house regulations.

Despite these challenges, comparisons between these disparate systems are considered crucial to the rendering of a holistic care concept. Only when both systems complement each other can comprehensive psychiatric care be generated in the German penal system.

## Conclusion

Although not every offender requiring psychiatric treatment needs to be referred to high-security hospital care, it should be noted that, in contrast to prison hospital psychiatry, these institutions provide a therapeutic environment suited to meet the specific needs of forensic psychiatric patients (34, 35, 66). It is therefore evident that prison psychiatry and the conditions of foreign national patients must be improved. To achieve this, the pre-trial assessment and (mental) health-related aspects of these patients should be further investigated.

There are numerous claims regarding the therapeutic conditions of ethnic-minority, immigrant, and foreign national prisoners, which should likewise apply to the psychiatric assessment of these groups (10, 13, 26, 45, 67). Schouler-Ocak and Aichberger (68) noted that despite wider acceptance among practitioners, the implementation of postulated adjustments—such as intercultural skills, native-speaking impartial interpreters, and regular supervision—remains arduous. It appears that societal structures and the healthcare system are unaware or incapable of coping with the unique requirements of ethnic-minority, immigrant, and foreign national patients, despite multiple publications postulating their relevance (20, 22, 27, 69).

Imprisoning seriously mentally ill patients means depriving them of adequate psychiatric treatment which is unlikely obtained in an environment known to trigger mental health problems by social isolation, sensory deprivation, physical inactivity, mental underload, and overcrowding (29, 70).

Furthermore, prison hospital psychiatry appears structurally incapable of implementing even the first of the Principles of Medical Ethics published by the United Nations (71) which postulates treatment “of the same quality and standard as is afforded to those who are not imprisoned or detained” (34, 70). According to Keppler et al. (70) prison health care does not adhere to approved quality standards such as consistent monitoring, timely implementation of modified treatment guidelines, and sufficient personnel and funding. Additionally, in contrast to high-security hospitals, prison hospital psychiatry lacks specific regulations relating to the admission, treatment, and discharge of patients (29, 33, 34).

As it may be reasonably assumed that insufficient treatment of patients inevitably leads to poor prospects, the overrepresentation of foreign national patients in German prison hospital psychiatry should be assessed critically. Due to rising immigration in recent years, cultural influences on mental health and delinquency are increasingly gaining in significance. Enhancing public and prison health care should not only be seen as a political duty; being responsive to the requirements of different minority groups also involves promoting the process of social integration, maintaining mental health, and preventing the aggravation of psychiatric symptoms (22, 72, 73).

## Data Availability Statement

The raw data supporting the conclusions of this article will be made available by the authors, without undue reservation, to any qualified researcher. Requests to access the Baden-Wurttemberg dataset should be directed to TR, t.ross@zfp-reichenau.de; requests to access the Berlin dataset should be directed to AO-W, annette.opitz-welke@charite.de.

## Ethics Statement

All data was obtained during routine administration and sufficiently anonymized. Approval for the research has been obtained from the Charité’s Ethics Committee.

## Author Contributions

AO-W, TR, and BN contributed to the conception and design of the study. AO-W and TR organized the databases. BN performed the statistical analysis. BN wrote the sections of the manuscript. All authors contributed to the revision of the manuscript, and they have read and approved the version submitted.

## Conflict of Interest

The authors declare that the research was conducted in the absence of any commercial or financial relationships that could be construed as a potential conflict of interest.
